# Applications of CAD/CAM Technology for Craniofacial Implants Placement and Manufacturing of Auricular Prostheses—Systematic Review

**DOI:** 10.3390/jcm12185950

**Published:** 2023-09-13

**Authors:** Waqas Tanveer, Angela Ridwan-Pramana, Pedro Molinero-Mourelle, Tymour Forouzanfar

**Affiliations:** 1 Department of Oral and Maxillofacial Surgery, Amsterdam University Medical Center, 1081 HV Amsterdam, The Netherlands; 2 Center for Special Care in Dentistry, Department of Maxillofacial Prosthodontics, Stichting Bijzondere Tandheelkunde, 1081 LA Amsterdam, The Netherlands; a.ridwan@amsterdamumc.nl; 3 Department of Reconstructive Dentistry and Gerodontology, School of Dental Medicine, University of Bern, CHE 3012 Bern, Switzerland; pedro.molineromourelle@zmk.unibe.ch; 4 Department of Oral and Maxillofacial Surgery, Leiden University Medical Center, 2333 ZA Leiden, The Netherlands; t.forouzanfar@lumc.nl

**Keywords:** auricular prosthesis, digital planning, surgical template, guided implant surgery, craniofacial implants

## Abstract

This systematic review was aimed at gathering the clinical and technical applications of CAD/CAM technology for craniofacial implant placement and processing of auricular prostheses based on clinical cases. According to the Preferred Reporting Items for Systematic Reviews and Meta-Analysis (PRISMA) guidelines, an electronic data search was performed. Human clinical studies utilizing digital planning, designing, and printing systems for craniofacial implant placement and processing of auricular prostheses for prosthetic rehabilitation of auricular defects were included. Following a data search, a total of 36 clinical human studies were included, which were digitally planned and executed through various virtual software to rehabilitate auricular defects. Preoperative data were collected mainly through computed tomography scans (CT scans) (55 cases); meanwhile, the most common laser scanners were the 3dMDface System (3dMD LLC, Atlanta, Georgia, USA) (6 cases) and the 3 Shape scanner (3 Shape, Copenhagen, Denmark) (6 cases). The most common digital design software are Mimics Software (Mimics Innovation Suite, Materialize, Leuven, Belgium) (18 cases), Freeform software (Freeform, NC, USA) (13 cases), and 3 Shape software (3 Shape, Copenhagen, Denmark) (12 cases). Surgical templates were designed and utilized in 35 cases to place 88 craniofacial implants in auricular defect areas. The most common craniofacial implants were Vistafix craniofacial implants (Entific Medical Systems, Goteborg, Sweden) in 22 cases. A surgical navigation system was used to place 20 craniofacial implants in the mastoid bone. Digital applications of CAD/CAM technology include, but are not limited to, study models, mirrored replicas of intact ears, molds, retentive attachments, customized implants, substructures, and silicone prostheses. The included studies demonstrated a predictable clinical outcome, reduced the patient’s visits, and completed the prosthetic rehabilitation in reasonable time and at reasonable cost. However, equipment costs and trained technical staff were highlighted as possible limitations to the use of CAD/CAM systems.

## 1. Introduction

Morphological deformity of the external ear, referred to as an auricular defect, can arise from surgical intervention following tumor resection, trauma, or congenital malformation [1,2,3,4]. There are two approaches to manage these defects: surgical reconstruction of the external ear or fabrication of auricular prostheses. Auricular prostheses are artificial, removable devices that replicate the morphology of the external ear and are customized to address the cosmetic and psychological challenges posed by auricular defects [3,4]. In principle, the surgical reconstruction of the ear is performed through the use of rib cartilage, which is carved intra-operatively to provide the auricular matrix, or by using a synthetic material framework [5]. Surgical reconstruction comprises multiple surgical revisions to obtain an acceptable outcome [6]. However, reconstruction of the ear through surgical procedures is generally difficult and often fails to provide a satisfactory outcome [3].

A failed autogenous reconstruction is one of the major indications for prosthetic rehabilitation, as fibrosis from previous surgeries makes it difficult to reconstruct the external ear surgically [7]. Prosthetic rehabilitation for auricular defects has been carried out for decades for cosmetic reasons; however, the development of the craniofacial implant (CI) was the milestone that provided optimum retention, support, and stability to the auricular prosthesis [8,9]. The bone thickness in the mastoid region varies between 2.5 mm and 5.5 mm; therefore, the length of craniofacial implants is usually selected between 3 mm and 5 mm to retain the auricular prostheses [10,11]. Upon osseointegration, CI can be used in combination with various types of retentive attachments (clip bar attachment, magnet attachment, locator attachment, or combination of these types of attachments, i.e., bar with locator attachments or bar with ERA attachment) to retain the auricular prostheses [11,12,13].

Successful auricular defect rehabilitation depends on comprehensive preoperative planning. The presence of mastoid air cells and the proximity of facial nerves and cranial structures make it challenging for maxillofacial surgeons [14]. The use of non-contact and non-invasive medical imaging (CT scan, CBCT, and MRI) has provided the solution to plan preoperatively and thereby prevent damage to the adjacent anatomical structures [14,15]. These imaging techniques provide detailed hard and soft tissue details to plan the precise location of implants in relation to the prosthesis [14,15]. Furthermore, laser scanners can record the surface details; however, the sharp groves and skin folds could be missing in these scan images [16]. CT scans can provide a complete image, but at the expense of X-ray radiation. Similarly, MRI can also provide complete images for 3D models; however, the resolution is low as compared to CT scan images.

Digital planning software have further accelerated the planning and designing stages of rehabilitation procedures in the last couple of decades [17,18]. This digital workflow requires the hard and soft tissue details of the affected and non-affected areas, which can be acquired through CT scans, MRIs, and laser scans [15]. The scanned images from radiographic techniques are in DICOM format, which is converted into STL format for modeling software [19]. Digital designing software uses these data to mirror the structures from the normal side to the defect side to construct the retentive attachments, molds, models, and surgical templates for implant placement [20,21]. Following the computer-aided design (CAD) step, physical models, molds, surgical templates, and even prostheses can be printed through the computer-aided manufacturing (CAM) process by using various materials, i.e., resins, acrylics, thermoplastic waxes, plastics, and metals [17,20,21].

Additionally, surgical navigation systems have added a dynamic approach to the digital workflow for rehabilitation procedures by enabling the surgeons to precisely control the position of instruments during the surgical procedures through multi-planner medical imaging views [10,22]. Once the navigation pointer touches the tissues on the surgical site, the virtual pointer recognizes the exact location on radiographic images, providing the surgeon with the ability to navigate through the anatomical structures while keeping the tract on the virtual anatomical map [22].

The cosmetic limitations of surgical auricular reconstruction paved the way for prosthetic rehabilitation by means of auricular prostheses [3,6]. In recent years, advances in digital technology, specifically Computer-Aided Design and Computer-Aided Manufacturing (CAD/CAM), have emerged as promising tools for enhancing preoperative planning, design, and fabrication of auricular prostheses. The application of digital technology for the fabrication of facial prostheses has reduced the patient’s visits, the clinical and laboratory time spent on each visit, and the steps of the fabrication of prostheses with a predictable outcome [23]. Patients can visualize the proposed plan before undergoing the surgical or prosthetic phases. Overall, CAD/CAM systems have been used for the preoperative planning, fabrication of surgical templates, models, molds, substructures, customized implants, and surgical navigation for prosthetic rehabilitation of auricular defects. This systematic review aims to comprehensively examine the clinical and technical applications of CAD/CAM technology in the preoperative planning, designing, and manufacturing processes of auricular prostheses for individuals with auricular defects. To our knowledge, while various studies have discussed the individual components of CAD/CAM technology and auricular prostheses, a comprehensive analysis of their integrated application in clinical practice remains scarce. By synthesizing available clinical data, this review seeks to offer insights into the effective utilization of CAD/CAM technology for enhancing prosthetic rehabilitation outcomes.

## 2. Materials and Methods

A systematic review was performed based on the protocol of Preferred Reporting Items for Systematic Reviews and Meta-Analyses (PRISMA) [24] to determine the PICO (patients (P), investigations (I), comparison (C), and outcomes (O)) question.

Patients: Patient having an auricular defect

Interventions: Applications of CAD/CAM-based systems for planning, designing, and manufacturing of auricular prostheses and craniofacial implant placement.

Comparison: Not applicable.

Outcome: Fabrication of auricular prostheses.

Therefore, the established question was adapted to the PIO question: “In patients with auricular defects (P), what are the technical and clinical applications of CAD/CAM technology for craniofacial implant placement (I) and the manufacturing of auricular prostheses (O)?”. This was performed while also considering that comparison (C) was not applicable in this systematic review.

### 2.1. Search Strategy

The electronic search was executed by using the following combinations of terms: (Prostheses AND Planning AND Guide).

Prosthesis: (auricular prostheses OR ear prostheses OR silicone auricular prosthesis) AND Planning: (software planning OR scanning OR CAD/CAM OR digital OR navigation OR 3D) AND Guide: (implants OR extraoral implants OR craniofacial implants OR surgical template OR surgical guide OR printed guide OR guided surgery OR navigation system).

### 2.2. Eligibility Criteria

**Figure 1:**
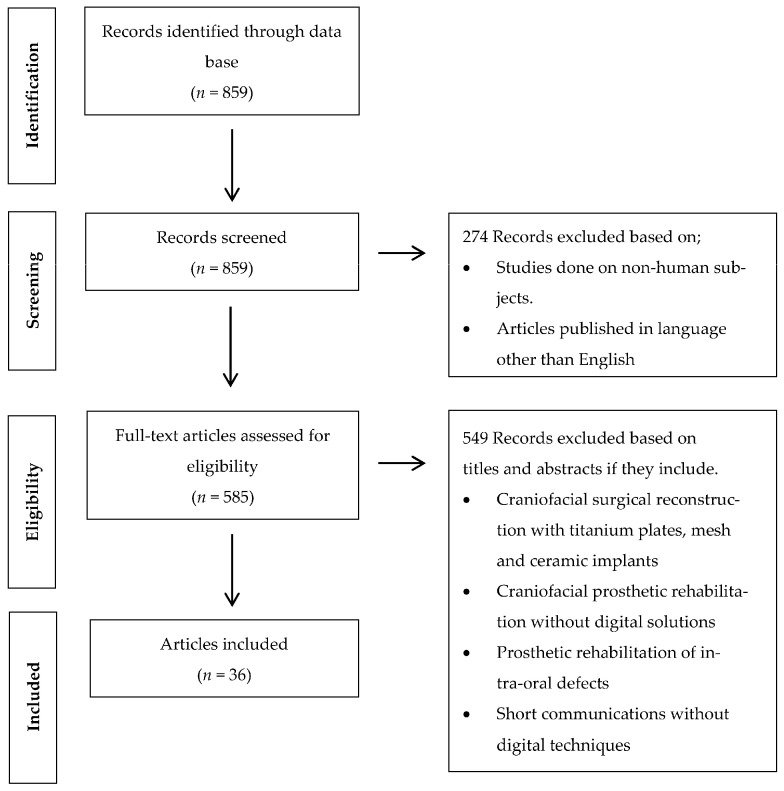
Flow chart of the study’s selection process and screening methodology.

Clinical human studies published in the English language between January 2000 and May 2023 were reviewed. The inclusion criteria were comprised of randomized control trials, case control studies, case reports, cohort studies, and case series utilizing digital software and CAD/CAM technology for orbital implant placement and manufacturing of auricular prostheses. The exclusion criteria included systematic reviews, animal studies, case reports, in vitro studies, and finite element analysis (FEA) studies executed without the use of digital software and CAD/CAM systems (Figure 1).

### 2.3. Source of Information

An electronic search was conducted for published articles between January 2000 and May 2023 in the National Library of Medicine (MEDLINE/PubMed) database.

Furthermore, a manual search of the published articles between January 2000 and May 2023 was also executed: The Journal of Prosthodontics, The Journal of Oral Rehabilitation, the International Journal of Prosthodontics, The Journal of Prosthetic Dentistry, The Journal of Oral Implantology, The Journal of Prosthodontic Research, The International Journal of Oral and Maxillofacial Implants, Clinical Oral Implants Research, International Journal of Oral and Maxillofacial Surgery, Journal of Stomatology, Journal of Oral and Maxillofacial Surgery, British Journal of Oral and Maxillofacial Surgery, Journal of Cranio-maxillo-facial surgery, Oral and Maxillofacial Surgery, Implant Dentistry, and Related Research.

### 2.4. Study Selection

The studies were reviewed by two independent reviewers (W.T. and P.M.M.) and selected on the basis of their titles and abstracts from the electronic search. Those studies that fulfilled the inclusion criteria or contained limited data in the abstract to reach the final decision were collected and reviewed. Disagreements among the authors were rectified after discussion.

### 2.5. Data Extraction

The useful data from the included studies were transferred to the standard designed form: authors, publication year, purpose of the digital planning, preoperative data, software used, printers utilized, materials for printing, number of implants placed, and implant systems in each case (Table 1). Authors were contacted for possible missing data.

### 2.6. Risk of Bias in Individual Studies

Two independent reviewers (W.T. and P.M.M.) evaluated the quality of the included studies. In case of a conflict of agreement regarding any publication, a third reviewer (A.R.P.) was contacted. For evaluation, the critical tools of the Joanna Briggs Institute [25] (JBI) for case series and case reports were utilized based on the type of included articles. The bias was evaluated on the basis of the list of 10 questions for case series and 8 questions for case reports, respectively. The questions listed in Table 2a–c and Table 3a,b concern the risk of bias. Eventually, an assessment was performed through an overall appraisal to determine if the risk of bias was low (inclusion of the study), high (exclusion of the study), or uncertain (more information was needed). We refer to it as a low risk of bias if the answer “yes” was ≥50%, an uncertain risk of bias if the answer “unclear” was ≥50%, and a high risk of bias if the answer “no” was ≥50%.

**Table 1:** Digital planning for the prosthetic rehabilitation of auricular defects.

Publications	No. of Cases	Purpose of Software Planning	Pre-Op Data for Digital Planning	Software	Printer/Miller	Printing Materials	Navigation System (Yes/No)	Location & No. of Implants	Implants System
Ciocca L., Scotti R. 2004 [26]	1	Fabrication of ear model	Minolta VIVID 900 3D laser scanner (Konica Minolta, Osaka, Japan)	Polygone editing tool (Minolta Co, Osaka, Japan), Rapidform CAD software (INUS Technology, Seoul, South Korea)	Z Printer 310 (Z Corp, Cambridge, MA, USA)	Powder and sealant from Z Corp. (Z Corp., Cambridge, MA, USA)	No	No	No
Sykes et al., 2004 [27]	1	Fabrication of ear model	Breuckmann OptoTOP scanner system (Breuckmann OptoTOP, Germany)	Polyworks software (InnovMetric Software), Freeform software (Freeform, NC, USA)	Thermojet printer	Wax material	No	No	No
Jiao et al., 2004 [28]	1	Fabrication of ear model	CT scan	Magics RP image ware (Materialise, Leuven, Belgium), Freeform software (Freeform, NC, USA)	Zippy-I RP machine (Kinergy Mechatronics, Singapore)	NM	No	No	No
Ciocca L et al., 2007 [29]	1	Fabrication of mold for auricular prosthesis and acrylic substructure	Minolta VIVID 900 3D laser scanner (Konica Minolta, Osaka, Japan)	Rapidform CAD software (INUS Technology, Seoul, South Korea), Software, Polygone editing tool (Minolta Co., Osaka, Japan)	Z Printer 310 (Z Corp, Cambridge, MA, USA)	Powder and sealant from Z Corp. (Z Corp., Cambridge, MA, USA)	No	No	No
Kurtulmus et al., 2009 [17]	1	Virtual implant planning	CT Scan	Implant 3D, Media Lab Software, 3D-Doctor software (Able Coorporation, Lexington, MA, USA)	NM	NM	No	No	No
Ciocca L et al., 2009 [30]	1	Surgical template for implant placement	CT, NextEngine Desktop 3D Scanner (NextEngine, Santa Monica, CA, USA)	Rapidform CAD software (INUS Technology, Seoul, South Korea)	Rapid prototyping machine (Z310Plus; Z Corp., Burlington, MA, USA)	NM	No	Right mastoid bone; 3 implants	NM
Turgut et al., 2009 [31]	10	Fabrication of ear model	CT scan	Modeling software (FreeForm Modeling Plus System, SensAble, Boston, MA)	Selective laser sintering (SLS) system (DTM Corp., Austin, TX, USA)	NM	No	No	No
Ciocca. L et al., 2010 [32]	1	Fabrication of mold for auricular prosthesis	NextEngine Desktop 3D Scanner (NextEngine, Santa Monica, CA, USA)	NextEngine Scan studio software (NextEngine, CA, USA)	3D printer (Stratasys, Eden Prairie, MN, USA)	ABS P400 jet (Stratasys, Eden Prairie, MN, USA)	No	No	No
Verma et al., 2010 [22]	2	Virtual planning and intraoperative navigation for implant placement	CT scan	Navigation system, Stryker iNtellect Cranial (Stryker Navigation system, MI, USA)	NM	NM	Yes	Left and Right mastoid regions; 4 implants	Vistafix implants (Cochlear, Lone Tree, USA)
De Crescenzio F et al., 2011 [33]	1	Fabrication of mold for auricular prosthesis	NextEngine Desktop 3D Scanner (NextEngine, Santa Monica, CA, USA)	Rapidform CAD software (INUS Technology, Seoul, South Korea), Rhinoceros Software v. 4.0 (Robert McNeel & Associates, USA)	3D printer (Stratasys, Eden Prairie, MN, USA)	ABS P400 jet (Stratasys, Eden Prairie, MN, USA)	No	Right mastoid bone; 2 implants	Straumann implants (Institut Straumann AG, Basel, Switzerland
Liacouras et al., 2011 [34]	1	Designing and creation of digital model and mold fabrication	CT scan, 3D photography/imaging (3dMD cranial System; 3dMD, Atlanta, GA, USA)	Mimics Software (Mimics Innovation Suite, Materialise, Leuven, Belgium), Freeform software (Freeform, NC, USA) (14 cases), Geomagics Studio software (3D Systems, Rock Hill, SC, USA)	ZPrinter 450, using zp130 Powder and zb59 Binder; (Z Corp., Cambridge, MA, USA)	NM	No	No	No
Kolodney et al., 2011 [35]	1	Surgical template for implant placement	CT scan	Mimics Software (Mimics Innovation Suite, Materialise, Leuven, Belgium), SurgiCase software (Materialise LLC, Ann Arbor, MI, USA)	NM	Somos DMX 100 Resin material (Somos DSM, Desotech Inc., Elgin, Illinois, USA)	No	Right mastoid bone; 3 implants	NM
Karatas MO et al., 2011 [36]	2	Fabrication of ear models	CT scans	Mimics Software (Mimics Innovation Suite, Materialise, Leuven, Belgium)	3D ink-jet FDM printer (Z Corp, Cambridge, MA, USA), Perfactory Standard SXGA+ stereolithography printer (Envisiontec Inc., Germany)	Acrylic material	No	No	No
Bai et al., 2012 [37]	1	Surgical template for implant placement	CT scan	Geomagics Studio software (3D Systems, Rock Hill, SC, USA), Mimics Software (Mimics Innovation Suite, Materialise, Leuven, Belgium)	Rapid Prototyping machine AFS-360 printer (Long yuan Technology Ltd., Beijing, China)	Resin material (Details NM)	No	Left mastoid region, 3 implants	NM
Reitemeier et al., 2012 [38]	1	Surgical template for implant placement	CT scan	Software (VoXim v6.1; IVS Solutions AG, Chemnitz, Germany)	FDM Vantage S; (Stratasys, Eden Prairie, MN, USA)	Acrylic resin template	No	Right mastoid region, 2 implants	Straumann implants (Straumann GmbH, Freiburg, Germany)
Hatamleh and Watson. 2013 [39]	1	Fabrication of ear model	3Shape R700 scanner (3 Shape, Copenhagen, Denmark)	3 Shape software (3 Shape, Copenhagen, Denmark)	Z-Corp Printer (Z Corp, Cambridge, MA, USA)	NM	No	2 craniofacial implants	Vistafix craniofacial implants (Cochlear, Surrey, UK)
Bai et al., 2014 [40]	6	Fabrication of mold for auricular prosthesis	CT scan, structured-light 3D scanner (3DSS-STD-II, Digital Manu, Shanghai, China)	Mimics Software (Mimics Innovation Suite, Materialise, Leuven, Belgium)	Rapid Prototyping machine AFS-360 printer (Long yuan Technology Ltd., Beijing, China)	Resin material	No	No	No
Tam CK et al., 2014 [8]	6	Fabrication of ear model	CT scan, 3dMDFace (3dMD, Atlanta, USA)	Mimics Software (Mimics Innovation Suite, Materialise, Leuven, Belgium), Surgical navigation system (BrainLAB, Feldkirchen, Germany)	Fused Deposition Modeling (FDM)	NM	Surgical navigation system (BrainLAB, Feldkirchen, Germany)	12 implants were placed in mastoid bone	Dental implants (Friadent, Dentsply, Mannheim, Germany)
Watson and Hatamleh 2014 [41]	3	Fabrication of ear model	3Shape R700 scanner (3 Shape, Copenhagen, Denmark)	Software Z-Build (Z Corp, Cambridge, MA, USA)	3D printer (Z Corp., Cambridge, MA, USA).	Gypsum (150 Powder) (Z Corp, Cambridge, MA, USA)	No	No	No
Wang et al., 2015 [42]	1	Fabrication of model for implant placement planning	EBCT scan	Geomagics Studio software (3D Systems, Rock Hill, SC, USA)	SLS machine (AFS-360; (Long yuan Technology Ltd., Beijing, China)	Resin material	No	3 implants in right mastoid bone	Implants (MDIC; FMMU, China)
Nuseir et al., 2015 [43]	1	Surgical template for implant placement	CT scan	Mimics Software (Mimics Innovation Suite, Materialise, Leuven, Belgium)	3D printer (Z Corp, Cambridge, MA, USA)	NM	No	Left mastoid region, 2 implants	Vistafix craniofacial implants (Cochlear, Surrey, UK
Choi et al., 2016 [44]	2	Planning for craniofacial implant placement	CT scan	BrainLAB software (BrainLAB AG, Munich, Germany)	No	No	Image guidance systems (IGS) (Brainlab AG, Munich, Germany).	4 implants in mastoid bone	Vistafix craniofacial implants (Cochlear, Surrey, UK)
Weissler et al., 2017 [45]	1	Virtual planning and intraoperative navigation for implant placement	CT scan, Laser scan	iPlan Cranial 3.0 BrainLAB software (BrainLAB AG, Munich, Germany)	NM	NM	Yes	Left & Right mastoid region; 4 implants	Vistafix craniofacial implants (Cochlear, Surrey, UK)
Yadav et al., 2017 [46]	1	Fabrication of mold for auricular prosthesis	CT scan	3D modeling software	NM	NM	No	No	No
Nafij Bin Jamayet et al., 2018 [47]	1	Fabrication of ear model	NextEngine Desktop 3D Scanner (NextEngine, Santa Monica, CA, USA)	NextEngine Scan studio software (NextEngine, CA, USA), Rapidworks 64 version 4.1.0. (3D system, Inc., Rock Hill, USA)	Objet30 Scholar 3D Printer (Stratasys, Eden Prairie, MN, USA)	NM	No	No	No
Unkovskiy et al., 2018 [48]	1	Fabrication of mold for auricular prosthesis	Artec Color 3D scanner (Artec 3D, Luxembourg)	Artec Studio Software (Artec 3D, Luxembourg), Z brush software (Pixologic, Inc., Los Angeles, CA, USA)	ProJet 3510 CPXPlus (3D Systems, Rock Hill, SC, USA), SPro 60 HD (3D Systems, Rock Hill, SC, USA)	VisiJet M3 Hi-Cast printer (3D Systems, Rock Hill, SC, USA)	No	No	No
Sanghavi, et al., 2018 [49]	1	Fabrication of ear model	CT scan	Freeform software (Freeform, NC, USA)	3D printing technology (Stereolithography)	Acrylic photopolymeric material	No	No	No
Ferreira R, Vives P. 2019 [50]	2	Fabrication of custom titanium plate for locator attachments	CT scan	Materialise 3-matic software 9.0 (Materalise, Leuven, Belgium)	Selective laser melting	Titanium grade 2	No	No	No
Vijverberg MA et al., 2019 [51]	11	Surgical template for implant placement	CT scan	3 Shape software (3 Shape, Copenhagen, Denmark)	NM	Polyamide material (Oceanz BV, Ede, The Netherlands)	No	31 VXI300 implants in mastoid bone	Vistafix implants (Cochlear Bone Anchored Solutions AB, Mölnlycke, Sweden)
Cevik and Kocacikli. 2020 [52]	1	Fabrication of mold for auricular prosthesis	Artec Color 3D scanner (Artec 3D, Luxembourg)	Artec studio 16 software (Artec 3D, Luxembourg)	FDM technology printer; MakerBot Replicator 2 (MakerBot Industries, Brooklyn, NY, USA)	Polylactic acid material (PLA)	No	No	No
McHutchion and Aalto. 2021 [53]	5	Fabrication of scan bodies and molds for auricular prosthesis	3dMD flex System (3dMD LLC, Atlanta, Georgia, USA), 3Shape R700 scanner (3 Shape, Copenhagen, Denmark)	Geomagics Studio software (3D Systems, Rock Hill, SC, USA)	Stereolithography 3D printer Form2 (Formlabs Inc., Somerville, Massachusetts, USA)	Clear resin, white resin (Formlabs Inc., Somerville, Massachusetts, USA)	No	No	No
Domingue D. et al., 2021 [54]	1	Surgical template for implant placement	CBCT scan	Meshmixer (Autodesk Inc., USA), Blue Sky Plan software (Blue Sky Bio, LLC, USA)	CEL Robox 3D printer (CEL, Bristol, UK)	nGen colorFabb polymer material (Eastman Chemical Company, Belfeld, Netherlands)	No	4 implants in right mastoid bone	Vistafix craniofacial implants (Cochlear, Surrey, UK)
Unkovskiy et al., 2021 [55]	1	Fabrication of ear model and substructure and printing of silicone auricular prosthesis	Pritiface 3D photogrammetry system (pritiface; pritidenta GmbH, Germany), 3Shape R700 scanner (3 Shape, Copenhagen, Denmark)	Exocad software (Exocad, GmbH, Darmstadt, Germany), Z brush software (Pixologic, Inc., USA)	Stereolithography (SLA) (Form 2; Formlabs) (Formlabs Inc., Somerville, Massachusetts, USA)	Resin material (Flexible; Formlabs) (Formlabs Inc., Somerville, Massachusetts, USA), ACEO silicone material (Drop-on-Demand ACEO; Wacker Chemie AG, Munich, Germany)	No	No	No
Dashti et al., 2022 [56]	1	Fabrication of working cast and bar	Artec Color 3D scanner (Artec 3D, Luxembourg), 3Shape R700 scanner (3 Shape, Copenhagen, Denmark)	Z brush software (Pixologic, Inc., USA), Exocad software (Exocad, GmbH, Darmstadt, Germany)	Stereolithography printer	PowerDent resin material (ProTech Transfer Co., Ltd., Bangkok, Thailand)	No	No	No
Hatamleh MM et al., 2022 [57]	3	Surgical template for implant placement	CT scan	CMF Pro Plan; Materialise	Form 2; Formlabs GmbH	NM	No	Patient 1: Right mastoid bone. 2 implants of 4 mm Patient 2: 2 implants on each side	Branemark; Cochlear Europe Ltd.
Heydenrych A et al., 2023 [58]	1	Surgical template for implant placement	CT scan	Materialise 3-matic software 9.0 (Materalise, Leuven, Belgium)	Selective laser sintering printer	Nylon PA 2200	No	Right mastoid bone. 3 implants	No

Abbreviations: CT: computed tomography; CBCT: cone-beam computed tomography; EBCT: electron beam computed tomography; Pre-op: preoperative; and NM: not mentioned.

**Table 2:** (a) Risk of bias for the case reports. (b) Risk of bias for the case reports. (c) Risk of bias for the case reports.

(**a**)									
**Assessment**	**Author and Year**								
	**Ciocca L, Scotti R. 2004** [26]	**Sykes et al., 2004** [27]	**Jiao et al., 2004** [28]	**Ciocca L et al., 2007** [29]	**Kurtulmus et al., 2009** [17]	**Ciocca L et al., 2009** [30]	**Ciocca. L et al., 2010** [32]	**De Crescenzio F et al., 2011** [33]	
Were patient’s demographic characteristics clearly described?	No	No	Yes	No	Yes	No	Yes	No	
Was the patient’s history clearly described and presented as a timeline?	No	No	Yes	No	Yes	No	Yes	No	
Was the current clinical condition of the patient on presentation clearly described?	No	Yes	Yes	No	Yes	Yes	Yes	Yes	
Were diagnostic tests or assessment methods and the results clearly described?	Unclear	Unclear	Unclear	Unclear	Unclear	Unclear	Unclear	Unclear	
Was the intervention(s) or treatment procedure(s) clearly described?	Yes	Yes	Yes	Yes	Yes	Yes	Yes	Yes	
Was the post-intervention clinical condition clearly described?	Yes	Yes	Yes	Yes	Yes	Yes	Yes	Yes	
Were adverse events (harms) or unanticipated events identified and described?	Yes	No	No	Yes	No	No	No	No	
Does the case report provide takeaway lessons?	Yes	Yes	Yes	Yes	Yes	Yes	Yes	Yes	
Overall appraisal	Included	Included	Included	Included	Included	Included	Included	Included	
(**b**)									
**Assessment**	**Author and Year**								
	**Liacouras et al., 2011** [34]	**Kolodney et al., 2011** [35]	**Bai et al., 2012** [37]	**Reitemeier et al., 2012** [38]	**Hatamleh and Watson. 2013** [39]	**Wang et al., 2015** [42]	**Nuseir et al., 2015** [43]	**Weissler et al., 2017** [45]	
Were patient’s demographic characteristics clearly described?	Yes	Yes	No	Yes	Yes	Yes	Yes	Yes	
Was the patient’s history clearly described and presented as a timeline?	Yes	Yes	No	Yes	Yes	Yes	Yes	Yes	
Was the current clinical condition of the patient on presentation clearly described?	Yes	Yes	No	Yes	Yes	Yes	Yes	Yes	
Were diagnostic tests or assessment methods and the results clearly described?	Unclear	Unclear	Unclear	Unclear	Unclear	Unclear	Unclear	Unclear	
Was the intervention(s) or treatment procedure(s) clearly described?	Yes	Yes	Yes	Yes	Yes	Yes	Yes	Yes	
Was the post-intervention clinical condition clearly described?	Yes	Yes	Yes	Yes	Yes	Yes	Yes	Yes	
Were adverse events (harms) or unanticipated events identified and described?	No	No	Yes	No	No	No	No	No	
Does the case report provide takeaway lessons?	Yes	Yes	Yes	Yes	Yes	No	Yes	Yes	
Overall appraisal	Included	Included	Included	Included	Included	Included	Included	Included	
(**c**)									
**Assessment**	**Author and Year**								
	**Yadav et al., 2017** [46]	**Nafij Bin Jamayet et al., 2018** [47]	**Unkovskiy et al., 2018** [48]	**Sanghavi, et al., 2018** [49]	**Cevik and Kocacikli. 2020** [52]	**Domingue D. et al., 2021** [54]	**Unkovskiy et al., 2021** [55]	**Dashti et al., 2022** [56]	**Heydenrych A et al., 2023** [58]
Were patient’s demographic characteristics clearly described?	Yes	No	Yes	Yes	Yes	Yes	No	No	No
Was the patient’s history clearly described and presented as a timeline?	Yes	No	Yes	Yes	Yes	Yes	No	No	No
Was the current clinical condition of the patient at presentation clearly described?	Yes	No	Yes	Yes	Yes	Yes	No	Yes	Yes
Were diagnostic tests or assessment methods and the results clearly described?	Unclear	Unclear	Unclear	Unclear	Unclear	Unclear	Unclear	Unclear	Yes
Was the intervention(s) or treatment procedure(s) clearly described?	Yes	Yes	Yes	Yes	Yes	Yes	Yes	Yes	Yes
Was the post-intervention clinical condition clearly described?	Yes	Yes	Yes	Yes	No	Yes	Yes	Yes	Yes
Were adverse events (harms) or unanticipated events identified and described?	No	Yes	No	No	No	No	Yes	Yes	No
Does the case report provide takeaway lessons?	Yes	Yes	Yes	Yes	Yes	Yes	Yes	Yes	Yes
Overall appraisal	Included	Included	Included	Included	Included	Included	Included	Included	Included

**Table 3:** (a) Risk of bias for the case series. (b) Risk of bias for the case series.

(**a**)							
**Assessment**	**Author and Year**						
	**Turgut et al., 2009 [31]**	**Verma et al., 2010 [22]**	**Karatas MO et al., 2011 [36]**	**Bai et al., 2014 [40]**	**Tam CK et al., 2014 [8]**	**Watson and Hatamleh 2014 [41]**	**Choi et al., 2016 [44]**
Were there clear criteria for inclusion in the case series?	Yes	Yes	Yes	Yes	Yes	Yes	Yes
Was the condition measured in a standard, reliable way for all participants included in the case series?	Yes	Yes	Yes	Yes	Yes	Yes	Yes
Were valid methods used for identification of the condition for all participants included in the case series?	Yes	Yes	Yes	Yes	Yes	Yes	Yes
Did the case series have consecutive inclusion of participants?	Unclear	Unclear	Unclear	Unclear	Unclear	Unclear	Unclear
Did the case series have complete inclusion of participants?	Yes	Yes	Yes	Yes	Yes	Yes	Yes
Was there clear reporting of the demographics of the participants in the study?	Yes	Yes	No	Yes	Yes	Yes	Yes
Was there clear reporting of clinical information of the participants?	No	Yes	Yes	Yes	Yes	No	Yes
Were the outcomes or follow-up results of cases clearly reported?	Yes	Yes	Yes	Yes	Yes	Yes	Yes
Was there clear reporting of the presenting site(s)/clinic(s) demographic information?	No	No	No	Yes	Yes	Yes	Yes
Overall appraisal	Included	Included	Included	Included	Included	Included	Included
(**b**)							
**Assessment**	**Author and Year**						
	**Ferreira R, Vives P. 2019** [50]	**Vijverberg MA et al., 2019** [51]	**McHutchion and Aalto. 2021** [53]		**Hatamleh MM et al., 2022** [57]		
Were there clear criteria for inclusion in the case series?	Yes	Yes	Yes		Yes		
Was the condition measured in a standard, reliable way for all participants included in the case series?	Yes	Yes	Yes		Yes		
Were valid methods used for identification of the condition for all participants included in the case series?	Yes	Yes	Yes		Yes		
Did the case series have consecutive inclusion of participants?	Unclear	Yes	Yes		Yes		
Did the case series have complete inclusion of participants?	Yes	Yes	Yes		Yes		
Was there clear reporting of the demographics of the participants in the study?	Yes	Yes	Yes		Yes		
Was there clear reporting of clinical information of the participants?	Yes	No	Yes		Yes		
Were the outcomes or follow up results of cases clearly reported?	Yes	Yes	Yes		Yes		
Was there clear reporting of the presenting site(s)/clinic(s) demographic information?	No	Yes	Yes		Yes		
Overall appraisal	Included	Included	Included		Included		

## 3. Results

### 3.1. Study Selection

The term was searched in the PubMed database. The literature search and the selection process have been summarized in Figure 1. Since most of the digital planning and designing developments have been noticed in the past two decades, the search strategy was decided to gather data within the time frame of January 2000 to May 2023, which yielded 806 studies. Two hundred and sixty-six (266) studies were excluded through the language (English) and human-based studies filters. Thereby, 540 studies were thoroughly screened based on their titles and abstracts in accordance with the inclusion and exclusion criteria, which led to the further exclusion of 504 studies on the basis of their study design and rehabilitation techniques (rehabilitation of auricular defects performed through surgical reconstruction and prosthetic rehabilitation performed through conventional procedures without digital planning and designing procedures). A total of 36 studies were included after reading full-text papers, which involved clinical cases digitally planned and processed for prosthetic rehabilitation of auricular defects. (Table 1) Due to the quality and data heterogeneity of the included studies, a meta-analysis could not be executed.

### 3.2. Study Characteristics

#### 3.2.1. CAD/CAM Technology Applications for Prosthetic and Surgical Purposes

The included studies discussed the following applications of digital technology for prosthetic rehabilitation of auricular defects: surgical templates (27 cases), fabrication of ear models (30 cases), fabrication of molds for silicone packing (17 cases), customized scan bodies (1 case), and custom titanium plates for locator attachments fabricated with grade 2 titanium (1 case). A surgical navigation system was used to place craniofacial implants for prosthetic rehabilitation of auricular defects (2 cases). 

#### 3.2.2. Preoperative Record for Digital Planning

Digital planning requires preoperative data for surgical and prosthetic procedures. The following modalities were used to gain virtual data for preoperative planning: non-contact medical images (CT scans, CBCT, and EBCT), laser scans, 3D structured light scans, and 3D photogrammetry systems.

Non-contact medical imaging systems: computed tomography scan (CT scan) (55 cases), cone-beam computed tomography scan (CBCT scan) (1 case), and an electron beam computed tomography scan (EBCT) (1 case). 

3D structured light scanning systems: 3dMDface System (3dMD LLC, Atlanta, GA, USA) (6 cases), 3dMD flex System (3dMD LLC, Atlanta, GA, USA) (5 cases) Artec Color 3D scanner (Artec 3D, Luxembourg) (3 cases), 3dMD cranial system (3dMD LLC, Atlanta, GA, USA) (1 case), and a Breuckmann OptoTOP scanner system (Breuckmann OptoTOP, Germany) (1 case).

Laser scanners: 3 Shape scanner (3 Shape, Copenhagen, Denmark) (6 cases), NextEngine Desktop 3D Scanner (NextEngine, Santa Monica, CA, USA) (4 cases), and a Minolta VIVID 900 3D laser scanner (Konica Minolta, Osaka, Japan) (2 cases). 

3D photogrammetry system: Pritiface 3D photogrammetry system (pritiface; pritidenta GmbH, Germany) (1 case).

#### 3.2.3. Preoperative Record for Digital Designing

The digital software utilized by the included studies were Mimics Software (Mimics Innovation Suite, Materialise, Leuven, Belgium) (18 cases), Freeform software (Freeform, NC, USA) (13 cases), 3 Shape software (3 Shape, Copenhagen, Denmark) (12 cases), Geomagics Studio software (3D Systems, Rock Hill, SC, USA) (6 cases), Rapidform CAD software (INUS Technology, Seoul, South Korea) (5 cases), Software Z-Build (Z Corp, Cambridge, MA, USA) (3 cases), Polygone editing tool (Minolta Co, Osaka, Japan) (2 cases), NextEngine Scan studio software (NextEngine, CA, USA) (2 cases), Magics Materialise software (2 cases), Artec studio software (Artec 3D, Luxembourg) (2 cases), Materialise 3-matic software 9.0 (Materalise, Leuven, Belgium) (3 cases), Z brush software (Pixologic, Inc., USA) (1 case), Exocad software (Exocad, GmbH, Darmstadt, Germany) (I case). Navigation system: Stryker iNtellect Cranial (Stryker Navigation system, MI, USA) (2 cases), and BrainLAB cranial navigation software (BrainLAB AG, Munich, Germany) (9 cases).

#### 3.2.4. Printing Systems Utilized for Surgical and Prosthetic Phases

Stereolithography (SLA), selective laser sintering (SLS), and fused deposition modeling (FDM) were the modalities used following the digital planning and designing phases to print the required models, molds, substructures, custom plates for retentive attachments, and surgical templates for craniofacial implants. 

Fused deposition modeling (FDM) printers: MakerBot Replicator 2 (MakerBot Industries, Brooklyn, NY, USA), Zprinter 450 (Z Corp., Cambridge, MA, USA), ZPrinter^®^ 310 Plus (Z Corp., Cambridge, MA, USA), Stratasys 400mc (Stratasys, Eden Prairie, MN, USA), 3D ink-jet Z printer (Z Corp., Cambridge, MA, USA), Stratasys FDM Vantage printer (Stratasys, Eden Prairie, MN, USA), Objet30 Scholar 3D Printer (Stratasys, Eden Prairie, MN, USA), and a CEL Robox 3D printer (CEL, Bristol, UK).

Selective laser sintering (SLS) printers: selective laser sintering (SLS) system (DTM Corp., Austin, TX), Zippy-I RP machine (Kinergy Mechatronics, Singapore), VisiJet M3 Hi-Cast printer (3D Systems, Rock Hill, SC, USA), and an AFS-360 printer (Long yuan Technology Ltd., Beijing, China). 

Stereolithography (SLA): Perfactory Standard SXGA+ stereolithography printer (Envisiontec Inc., Germany), and a Form2 printer (Formlabs Inc., Somerville, MA, USA).

Printing materials: Z-corp powder sealant material (Z Corp, Cambridge, MA, USA), Acrylonitrile butadiene styrene plastic material ABS—P400 Jet (Stratasys, Eden Prairie, MN, USA), Polyamide material (Oceanz BV, Ede, The Netherlands), Clear Resin, white resin (Form2labs) (Formlabs Inc, Somerville, MA, USA), Somos DMX 100 Resin material (Somos DSM, Desotech Inc, Elgin, IL, USA), nGen colorFabb polymer material (Eastman Chemical Company, Belfeld, The Netherlands), PowerDent resin material (ProTech Transfer Co. Ltd., Bangkok, Thailand), Polylactic acid material (PLA) (MakerBot Industries, Brooklyn, NY, USA), and a Nylon PA 2200 (3DPRINTUK, London, UK).

#### 3.2.5. Guided Implant Surgery

A total of 88 craniofacial implants for auricular defects were placed in 77 clinical cases after the digital planning, designing, and manufacturing of surgical templates. A total of 51 Vistafix craniofacial implants (Entific Medical Systems, Goteborg, Sweden) were placed in 22 clinical cases, 3 implants (MDIC; FMMU, China) were placed in 1 case, 4 Straumann implants (Straumann GmbH, Freiburg, Germany) were placed in 2 cases, and 12 dental implants (Friadent, Dentsply, Mannheim, Germany) were placed in 6 cases, respectively, to rehabilitate with silicone auricular prostheses. Meanwhile, 20 implants were placed with the help of surgical navigation systems: Stryker iNtellect Cranial (Stryker Navigation system, MI, USA) and BrainLAB software (BrainLAB AG, Munich, Germany) to rehabilitate 9 patients with auricular prostheses. Only one study mentioned the postoperative accuracy of 3D-planned implant placement. According to the results, 3 implants were deviated by 3.814 mm, 5.747 mm, and 4.463 mm, respectively, with mean a value of 4.675 mm. 

### 3.3. Risks of Bias in Individual Studies

The JBI criteria were followed to assess the risk of bias in the individual studies. As illustrated by Table 2a–c, the case reports were authored by the following: Ciocca L, Scotti R. 2004 [26], Sykes et al., 2004 [27], Jiao et al., 2004 [28], Ciocca L et al., 2007 [29], Kurtulmus et al., 2009 [17], Ciocca L et al., 2009 [30], Ciocca. L et al., 2010 [32], De Crescenzio F et al., 2011 [33], Liacouras et al., 2011 [34], Kolodney et al., 2011 [35], Bai et al., 2012 [37], Reitemeier et al., 2012 [38], Hatamleh and Watson 2013 [39], Wang et al., 2015 [42], Nuseir et al., 2015 [43], Weissler et al., 2017 [45], Yadav et al., 2017 [46], Nafij Bin Jamayet et al., 2018 [47], Unkovskiy et al., 2018 [48], Sanghavi, et al., 2018 [49], Cevik and Kocacikli 2020 [52], Domingue D. et al., 2021 [54], Unkovskiy et al., 2021 [55], Dashti et al., 2022 [56], Heydenrych A et al., 2023 [58] showed a low risk of bias. Meanwhile, Table 3a,b showed that the case series authored by Turgut et al., 2009 [31], Verma et al., 2010 [22], Karatas MO et al., 2011 [36], Bai et al., 2014 [40], Tam CK et al., 2014 [8], Watson and Hatamleh 2014 [41], Choi et al., 2016 [44], Ferreira R, Vives P 2019 [50], Vijverberg MA et al., 2019 [51], McHutchion and Aalto 2021 [53], and Hatamleh MM et al., 2022 [57] presented a low risk of bias.

**Figure 2:**
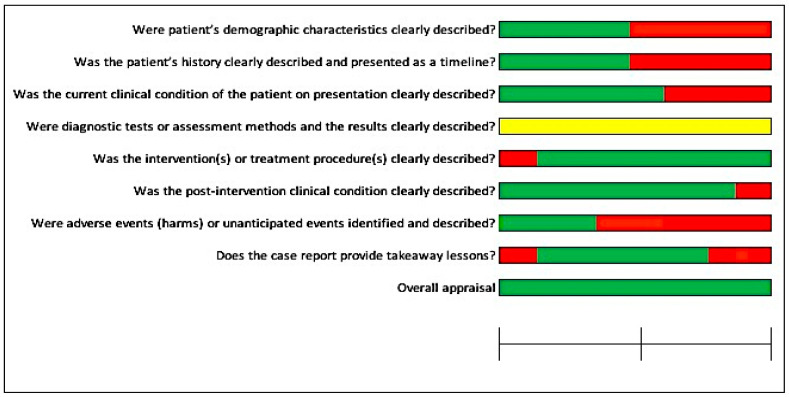
Risk of bias across the included studies for case reports.

In Figure 2, most studies had a low risk of bias (≤50%), except for the specific question, “Were adverse events (harms) or unanticipated events identified and described?”, where more than 75% of the included studies had not mentioned any adverse events or unanticipated events. For another question, “Were the diagnostic tests or assessment methods and the results clearly described?”, more than 75% of the studies had not clearly mentioned the diagnostic tests, assessment methods, or results of the investigations.

**Figure 3:**
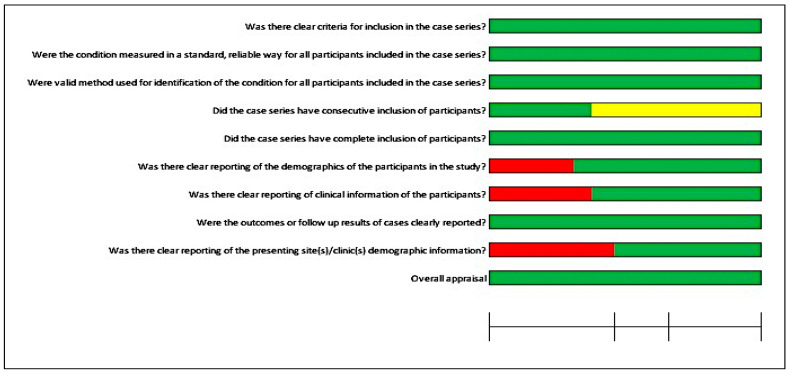
Risk of bias for the case series.

Furthermore, Figure 3 illustrates the risk of bias for four case series studies. Most questions were in favor of a low risk of bias. For two questions, the details were unclear: “Were valid methods used for identification of the condition for all participants included in the case series?” and “Was there clear reporting of clinical information of the participants?”. Furthermore, it was not possible to perform a meta-analysis due to the quality of the included studies, case series, and case reports.

## 4. Discussion

Digitally assisted design and digitally assisted manufacturing (CAD/CAM) systems have been utilized for the design and manufacturing of medical devices for the last couple of decades. The digital planning software were first utilized for intraoral implant placement in 1997 [59,60]. Further digital and technical advancements led clinicians and dental technologists to plan guided implant surgeries, the manufacturing of custom implants, retentive attachments, digital wax-ups, molds, and prostheses [61,62]. With the CAD/CAM applications, virtual surgical planning and its application in surgical procedures became more predictable, reduced the laboratory and clinical time for the procedures, reduced the patient’s appointments, and enabled the patients to virtually observe the proposed outcome prior to invasive procedures [61,62]. Various clinical case studies have documented the applications of digital technology for the fabrication of auricular prostheses; therefore, the aim of this paper was to gather the clinical studies involving the clinical and technical applications of CAD/CAM technology for craniofacial implant placement and the fabrication of auricular prostheses.

Three-dimensional imaging has added an extra dimension to the conventionally available preoperative radiographs, with the additional advantage of low radiation doses and detailed information about the bone quantity, bone volume, and proximity of adjacent critical anatomical structures [63,64]. The obtained data from computed tomography (CT), magnetic resonance imaging (MRI), or cone-beam computed tomography (CBCT) can be used in conjunction with digital planning software for preoperative planning [65,66]. The obtained data from tomographic images in combination with digital planning software help guide the implant placement in the optimum position and angulation according to the surgical and prosthetic plan [65,67]. Various factors such as tube current, slice thickness, voltage, pitch, the reconstruction algorithm for image slices, minor patient movement, and artifacts from metal objects can induce significant errors [68]. Among these factors, the slice thickness can influence the volume measurement, thus it should be set at <1.25 mm [68,69]. A total of seven included studies mentioned the slice thickness of CT scans ranging from 1 to 1.25 mm [28,34,35,37,40,44,49], while two included studies made use of a slice thickness less than 1 mm [31,50]. Furthermore, the voxel size affects the quality and reconstruction time of the CBCT images. None of the included clinical studies mentioned the voxel size.

The integration of 3D radiographic images and laser scans enabled the preoperative planning for guided implant surgeries. These two entities, when incorporated into the digital designing software, provided the possibility for maxillofacial surgeons and prosthodontists to plan the surgeries in chronological sequence (prosthesis-driven implant placement), from prosthetic design and position downwards to the implant position and angulation [70]. In this study, 77 cases were planned and executed by utilizing CT scans, CBCT scans, EBCT scans, MRIs, and laser scanners; however, only 14 cases were planned by the combined use of 3D radiographic images and laser scans for preoperative planning and designing of auricular prostheses.

Virtual planning has been mainly dependent on computer-aided design systems (CAD). These designing systems combine laser scans of intact and defect sides as well as 3D tomographic images to estimate the exact location and angulation of implants, to design the surgical templates, to plan and design the retentive attachments, and to design the molds, frameworks, customized implants, and provisional and definitive prostheses. In the current study, Meshmixer (Autodesk Inc., USA), Geomagics Studio software (3D Systems, Rock Hill, SC, USA), 3 Shape software (3 Shape, Copenhagen, Denmark), iPlan Cranial 3.0 BrainLAB software (BrainLAB AG, Munich, Germany), BrainLAB software (BrainLAB AG, Munich, Germany), Mimics Software (Mimics Innovation Suite, Materialise, Leuven, Belgium), Rapidform CAD software (INUS Technology, Seoul, South Korea), Rhinoceros Software v. 4.0 (Robert McNeel & Associates, USA), and Stryker iNtellect Cranial (Stryker Navigation system, MI, USA) were used in a total of 77 cases to plan and place 88 implants in auricular defects for prosthetic purposes. These implants were guided by surgical templates to be placed between 9 and 11 o’clock positions on the right side and between 1 and 3 o’clock positions on the left side, respectively [10]. Meanwhile, Z brush software (Pixologic, Inc., USA), Exocad software (Exocad, GmbH, Darmstadt, Germany), Geomagics Studio software (3D Systems, Rock Hill, SC, USA), Artec studio 16 software (Artec 3D, Luxembourg), Materialise 3-matic software 9.0 (Materalise, Leuven, Belgium), Freeform software (Freeform, NC, USA), NextEngine Scan studio software (NextEngine, CA, USA), Rapidworks 64 version 4.1.0. (3D system, Inc. Rock Hill, USA), Mimics Software (Mimics Innovation Suite, Materialise, Leuven, Belgium), and Polygone editing tool (Minolta Co, Osaka, Japan) designing software were used in 42 cases to design the scan bodies, customized implants, retentive attachments, models, molds, substructures for silicone auricular prostheses.

The computer-aided designing (CAD) step ultimately leads to the computer-aided manufacturing (CAM) step in order to convert the virtually planned and designed models, molds, templates, and prostheses to physical form by utilizing 3D printing systems [71,72,73]. Currently, six prototyping technologies can be used to convert virtually planned and designed objects into physical reality: stereolithography, laminated object manufacturing, selective laser sintering, solid ground curing, 3D ink-jet printing, and fused deposition modeling [36]. However, stereolithography (SLA), selective laser sintering (SLS), and fused deposition modeling (FDM) are the most frequently used 3D printing technologies. The FDM technology makes use of plastic filament, which is heated and extruded through the extrusion head onto the platform. As soon as extruded filament drops on the platform, it hardens due to the controlled temperature. In this way, layer-by-layer deposition builds up a physical model. To construct more complex models, multiple extrusion heads are required [73]. Plastics used for FDM technology are mainly acrylonitrile butyric styrene (ABS), polycarbonates, and polysulfides. The SLA technology utilizes ultraviolet light to polymerize the photosensitive resin. Following each layer of resin deposition on the platform, ultraviolet light cures successive layers, and photopolymerization helps to build up complex structures [73]. SLA-based printing technology utilizes a monomer resin that converts into a polymer upon photopolymerization. FDM printers are usually used to print models, molds, and provisional prostheses, while SLA printers are mainly used to print surgical templates for guided implant placement surgeries [74]. In the current review, SLA printing technology was used for 10 cases, SLS printers were used for 22 cases, and FDM printers were used for 40 cases. The most common printing materials were resin powders, polylactic acid (PLA), polyamide, titanium grade 2, gypsum powder, and ABS material.

Craniofacial implants were virtually planned for precise placement in the mastoid bone for the support and retention of auricular prostheses. A total of 88 implants were placed in right and left auricular defects in 35 cases following digital planning. Surgical templates and navigation systems were used to guide the implants in the planned locations. Due to various factors such as the anatomical morphology, radiation therapy, chemotherapy, and morphology of the tissue bed, two to four implants were placed in each auricular defect for prosthetic rehabilitation. Retention of auricular prostheses was mainly gained by clip bar and magnet attachments; however, locator attachment with a customized bone plate was also reported in one study [50]. Postoperative data to assess the accuracy of digitally planned extraoral implant placement for facial prosthetic rehabilitation are very limited [61,62]. Only one included study mentioned the postoperative accuracy of digitally planned auricular implant placement. Three implants had deviated by an average of 4.67 mm. However, the case was successfully rehabilitated by using an orientation guide for an auricular prosthesis [58].

**Figure 4:**
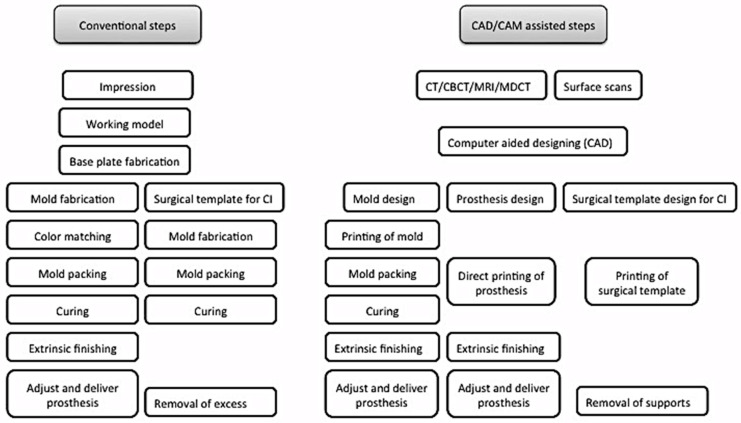
Comparison between conventional and digital planning and designing of surgical templates and prostheses fabrication.

Digital planning and design systems have reduced the patient’s visits to a minimum of two to three visits. [29,33,40] Table 4. Furthermore the included studies showed satisfactory clinical outcome for the prosthetic rehabilitation of auricular defects (Table 5). Mirroring the intact ear to the defect side helps to obtain the digital model, which can be printed to replicate the ear wax pattern [27,28,29,32,33,40,41]. The time required to plan, design, and manufacture the wax pattern through CAD/CAM systems ranged from 40 min to 4 h, which if processed conventionally would require more than 6 h [27,41] (Table 4). The systematic reviews from Tanveer, W. et al. [61,62] further provided the expected time and cost for the digital workflow involved in the processing of facial prostheses for the readers to obtain a general overview of the time and cost involved in the digital planning, designing, and manufacturing of facial prostheses. Navigation systems have further paced the surgical planning and placement of implants. According to Verma et al., 2010 [22], the navigation system for guiding the craniofacial implants had reduced the clinical and laboratory time by 10 h. Additionally, digital workflow has enabled patients to visualize the proposed plan prior to invasive procedures, thereby giving the option to the patients to either accept or reject the proposed plan based on the expected outcome. Once the clinician and patient decide to proceed, the planning and designing software helps to construct surgical templates, models, molds, customized implants, retentive attachments, and even direct silicone prostheses (Figure 4).

The current systematic review includes clinical studies that were planned and executed from data acquisition to the virtual designing and printing of surgical templates, molds, substructures, implants, retentive attachments, and auricular prostheses. CAD/CAM systems have provided numerous advantages over conventional processing, such as predictability, reduced clinical and laboratory time, reduced patient visits, and the ability to view and discuss the end outcome before invasive procedures. However, studies about the accuracy of these digital planning software and printing systems are not yet available. Therefore, clinical trials are needed to assess the precision and accuracy of these CAD/CAM systems, especially for guided implant surgeries. Furthermore, the cost of equipment, maintenance, and trained technical staff pose limitations; therefore, these facilities are only accessible in high-end centers. Printing of color-matched prostheses, direct printing of prostheses with medical-grade silicone, and controlled fine thickness of the margins of prostheses are the other limitations that need to be addressed with further digital and technical advancements.

## 5. Conclusions

CAD/CAM systems have been used for maxillofacial prosthetics in the planning, designing, and manufacturing stages for the last couple of decades. Clinical and technical applications of CAD/CAM technology include data acquisition, planning and designing surgical templates, models, molds, retentive attachments, customized implants, and the manufacturing of prostheses. These CAD/CAM systems have shown a predictable clinical outcome, reduced the clinical and technical time to fabricate auricular prostheses, and reduced the patient’s appointments when compared to conventional processing techniques. However, the availability of trained technical staff and the equipment cost limit the use of CAD/CAM in most parts of the world. Despite the digital advancements, direct printing of silicone auricular prostheses, production of featheredge thin margins, and direct printing of color-matched prostheses are the few current limitations of CAD/CAM-assisted techniques that need to be addressed. Furthermore, human clinical trials are needed to determine the precision and accuracy of these CAD/CAM systems for craniofacial implant placement and the fabrication of auricular prostheses.

## Figures and Tables

**Table 4:** Efficiency of digital workflow verses conventional processing of auricular prostheses.

Studies	Purpose	Digital Process				Convencional Process
		Material	Cost	Time	No. of Appointments	Time
Sykes et al., 2004 [27]	Digitization of ear model and processing in Freeform software for rapid prototyping of mirrored ear model	Wax	NM	4 h	NM	6 h
Jiao et al., 2004 [28]	Printing and finishing of ear prosthesis	NM	NM	1.5 h	NM	NM
Ciocca L et al., 2007 [29]	Computer-aided design and rapid prototyping of auricular mold and substructure	Resin	15$	NM	3	NM
Ciocca. L et al., 2010 [32]	Computer-aided design and rapid prototyping of auricular molds and substructures for bilateral auricular prostheses	—ABS	36.58€	10 h 42 min	NM	NM
De Crescenzio F et al., 2011 [33]	Computer -aided design and rapid prototyping of auricular mold and substructure	ABS	23.79€	6 h 39 min	3	NM
Watson and Hatamleh 2014 [41]	Scanning, manipulation, RP build, and finishing time for wax prototype	Wax	58$	40 min	NM	2 h
Bai et al., 2014 [40]	Clinical time spentComputer-aided design and rapid prototyping of auricular molds	Resin	NM	4 h10 h	2	NM

Abbreviations: NM—Not mentioned; H—hour; Mins—minutes.

**Table 5:** Enlisted are the clinical outcomes, recommendations, and limitations mentioned in the included clinical studies.

Included Articles	Outcome	Recommendations	Limitations
Ciocca L, Scotti R. 2004 [26]	The stone cast of existing ears was scanned and mirrored with the help of software to the affected side. A 3D printer helped print the resin model of the ear for the affected side.	The technique utilized in this clinical case is faster; it takes about 8 h. Once the equipment is available, the cost of individual procedures can be less expensive as compared to the anaplastologist’s work.	The limitation of this clinical technique is the inability to reciprocate the color information, and the equipment cost can be expensive.
Sykes et al., 2004 [27]	Digital technology provided the way to obtain an accurate wax model in a reasonable short time as compared to conventional methodology for the rehabilitation of auricular defects.	External imaging methods can provide better accuracy than CT scan images, especially in cases of complex ear anatomy. Additionally, it does not require volumetric modeling interpolation between the slices.	High cost of equipment and a trained laboratory technician for digital designing and printing procedures.
Jiao et al., 2004 [28]	The shape, dimension, and anatomic contour of the ear prosthesis matched the normal side and fitted well on the defect side.	The Magics RP (Materilise) program permits the mirrored ear position to move anteroposterior superioinferior, projections from the surface to match the unaffected side precisely.	According to the authors, improvement of the technique is required.
Ciocca L et al., 2007 [29]	The described protocol enabled the fabrication of an ear prosthesis in three appointments through the construction of a virtual CAD/CAM model, a mold, and digitization of the implant location.	This protocol allows the scanning and replacement of a lost ear virtually without the need for a diagnostic wax pattern.	The limitations are the technical skills required to use CAD/CAM technology and the relevant costs of laboratory equipment, including 3D scanners and rapid prototyping machines.
Kurtulmus et al., 2009 [17]	The prosthetically driven implant’s location was determined by mirroring the normal ear to the defect side and the virtual construction of the model. The prosthesis was processed and fitted in a conventional way.	NM	NM
Ciocca L et al., 2009 [30]	The prosthetically driven implant’s locations were determined. A surgical template was printed to place the implants in the planned locations.	The location of landmarks during a CT scan and the duplication of the diagnostic template can induce errors; therefore, the authors recommend their protocol for the correct diagnosis of available bone and the precise transfer of planning to the surgical template.	NM
Turgut et al., 2009 [31]	Auricular prostheses were manufactured through mirroring of the contralateral normal ear and rapid prototyping of a 3D ear model. Digital technology helped complete the prosthetic rehabilitation with only one trial session.	The auricular prosthesis procedure was cost-effective, required fewer patient visits, and eliminated the chances of sculpturing errors by utilizing rapid prototyping methodologies.	The protocol described here is only applicable to patients with one missing ear. According to the authors, in the case of bilateral auricular atresia, esthetic ratios of facial features can be used as a guideline to design prostheses.
Ciocca. L et al., 2010 [32]	Bilateral auricular defects were rehabilitated by selecting a reference ear from the digital ear and nose library. The mold and substructure were designed virtually and rapidly prototyped for silicone packing to obtain bilateral auricular prostheses.	The Ear and Nose Digital Library is a useful tool if the patient has bilateral auricular defects. Mirroring a selected reference ear from a digital library can provide identical bilateral auricular prostheses.	The presence of a stair-case effect on the superficial surface is one limitation that can be minimized by using thin printing layers or using surface finishers conventionally, following construction of models or molds.
Verma et al., 2010 [22]	A surgical navigation system was used to plan and place the craniofacial implants in two patients without any reported complications. Prostheses were fabricated in a conventional way.	The authors recommend this technique as it is virtually possible to visualize future prostheses, which helps surgeons alter the soft tissues prior to or during surgery without the necessity to keep a soft tissue profile for soft tissue registered surgical templates.	NM
De Crescenzio F et al., 2011 [33]	A three-piece mold was designed and 3D-constructed by means of CAD/CAM technology. Precise positioning of the substructure was attained in the prototype mold. Molds are fitted precisely with silicone packing.	This technique reduced the time and cost considerably when compared to conventional procedures and anaplastologist services. To reduce the stair-case effect, mold parts were oriented along the vertical axis of the prosthesis (from bottom to top).	NM
Liacouras et al., 2011 [34]	A mold for an auricular prosthesis was designed and printed using rapid prototyping technology for silicone packing. A three-piece mold fitted well to the fabricated silicone auricular prosthesis.	Rapid prototyping material is usually devoid of color; therefore, it prevents contamination, which is the issue with colored stone molds. A three-piece mold is recommended for ease of recovery, positioning of the seam at a favorable spot, and ease of placement of the retentive substructure.	Laboratory technicians can induce very limited surface texture in 3D printed molds; therefore, it is difficult to produce that surface texture in a finished prosthesis. Furthermore, the cost and technical skills required to use CAD/CAM technology are the limiting factors.
Kolodney et al., 2011 [35]	Cephalometric analysis was used to plan the implant’s locations digitally and transferred into a physical ear model by rapid prototyping. The planned implant locations were then copied into a surgical template for craniofacial implant placement.	The authors recommend this technique of using cephalometric analysis to locate the implant’s location in patients who are facially symmetric. It is not applicable to craniofacial anomaly cases.	Despite the advantages of this technique, it is still necessary to place the physical wax pattern on the patient to verify the shape and position before proceeding to process it with silicone to fabricate a prosthesis.
Karatas MO et al., 2011 [36]	Intact ears were mirrored onto the defect side. Rapid prototyping technology was used to construct the ear models. Following the duplication of models, auricular prostheses were fabricated in a conventional way.	The authors suggest scanning and mirroring the intact side to the defect side, as these steps eliminate the potential errors from impression and wax carving.	The skilled laboratory technologist and cost of designing and printing equipment pose limitations to this technique.
Bai et al., 2012 [37]	The surgical template was digitally designed to place the auricular implants. A maxillary occlusal splint was connected to a surgical template to stabilize it, and implants were placed through flapless surgery.	The authors recommend this technique to design the surgical template for implant placement as it permits flapless surgery and precise implant placement, reducing the procedure time and morbidity.	This technique can only be used on dentate patients, as the surgical template needs to be stabilized with an occlusal splint.
Reitemeier et al., 2012 [38]	The surgical template for implant placement was designed by mirroring the intact ear to the defect side. The glabella and nose were used to stabilize the surgical template during the surgical procedure.	This approach facilitates the design of a surgical template for implant placement, which guides the symmetrical positioning of an auricular prosthesis.	The additional cost of a surgical template for implant placement can pose a limitation to this technique.
Hatamleh and Watson. 2013 [39]	The surgical template for implant placement was designed by mirroring the intact ear to the defect side. The ear model was printed and duplicated to adapt to the working cast for precise positioning during implant placement. Two implants were placed at the planned position in the mastoid bone.	Digital mirroring and printing the ear saved time, which is usually spent on wax carving. Mirrored images were saved in a computer system, which can be used to reprint the ear model for future prostheses, thus saving the patient’s visit and storage space.	NM
Bai et al., 2014 [40]	Intact ears were mirrored on the defect side and used to create a negative three-piece mold for silicone packing. Prostheses fitted well in all cases, and patients showed thorough satisfaction.	With this technique, the try-in step can be eliminated, thereby reducing the patient’s visits. Furthermore, by eliminating manual flasking and investing procedures. The overall time of fabrication was significantly reduced.	An objective investigation of patient satisfaction is required to evaluate the acceptance of prostheses following this digital technique.
Tam CK et al., 2014 [8]	The intact ear was mirrored on the defect side to obtain the prototyped model. A surgical navigation system was utilized to place the craniofacial implants. The delivered prostheses had good retention, stability, and a symmetrical position. Psychological assessment showed decreased depressive symptoms and positive emotions.	Computer-assisted planning and surgical navigation systems have been recommended by the authors. CAD software helps in preoperative planning, while surgical navigation systems improve intraoperative safety and prevent damage to critical anatomic structures.	The authors encountered problems with reduced retention of prostheses and discoloration or color mismatch of silicone auricular prostheses.
Watson and Hatamleh 2014 [41]	The intact ear was mirrored on the defect side and prototyped to obtain a 3D model. The obtained model was copied into a wax pattern and adjusted in a trial session on the patient. Mirroring the ear model produced an accurate shape and form, comparable to the intact normal ear.	The presented technique required less clinic time. Scanning, manipulation, rapid prototyping, and finishing the wax prototype took 40 min, while an average ear wax pattern requires 2 h.	The limitation of this technique is the cost of software and hardware use, maintenance, and trained staff to operate these digital systems.
Wang et al., 2015 [42]	The normal ear was mirrored on the defect side, and a surgical template was designed to place three implants on the defect side. The ear model was printed and duplicated into a wax pattern for trial and laboratory processing.	Remnants of the malformed ear should be removed to facilitate the correct position and enhance the stability of the prosthesis.	NM
Nuseir et al., 2015 [43]	The normal ear was mirrored on the defect side, and a surgical template was designed to place two implants on the defect side. The ear model was printed and duplicated into a wax pattern for trial and laboratory processing.	This technique saved time and reduced the patient’s visits. Additionally, current data would be useful for future fabrication of prostheses without the patient’s physical presence.	NM
Choi et al., 2016 [44]	An intraoperative navigation system was used to place two craniofacial implants at the auricular defect location. The trajectory of the implants was confirmed at the planned location by using a navigation probe. An implant-retained ear prosthesis was fabricated without complications.	The intraoperative navigation system provides real-time navigation into the localized anatomy on the backdrop of multiplanar views. This system is recommended by the authors for patients with altered anatomy and limited bone availability.	According to the authors, this study is limited by the number of cases and the study design. The goal of this study was to highlight management principles for patients with altered anatomy only.
Weissler et al., 2017 [45]	The father’s ear was scanned and mirrored on the defect side for implant planning. An intraoperative navigation system was used to place three implants bilaterally, while two implants on each side were used for prostheses retention without any reported complications.	The intraoperative navigation system helps to execute the planned cases precisely, especially in failed auricular reconstruction attempts or complex cases with limited bone availability.	NM
Yadav et al., 2017 [46]	The normal ear was mirrored on the defect side and used to design the mold for silicone packing. The prosthesis recovered from a 3D-printed mold was adjusted and delivered. The patient was satisfied with the final outcome.	The scanning of soft tissues prevents the distortion that is inevitable with conventional impression techniques. A digitally designed prosthesis is a more accurate procedure. The mold prepared by using this reported technique can be used multiple times to pack silicone.	The technique used in this case requires expensive equipment and a trained computer graphic designer, which ultimately increases the cost of a prosthesis.
Nafij Bin Jamayet et al., 2018 [47]	The intact ear was mirrored on the defect side and printed to obtain a 3D ear model. The model was duplicated into a wax pattern and processed with conventional techniques to fabricate an auricular prosthesis.	The digital system used in this study was portable, easy to use, saved clinic time, and produced an exact replica of a normal ear for the defect side.	According to the authors, this technique lacks the ability to procedure silicone prostheses with proper color match with adjacent skin.
Unkovskiy et al., 2018 [48]	The normal ear was mirrored on the defect side and processed with two different techniques: direct mold making (DMM) and indirect mold making (IMM). The authors concluded that IMM is the preferred technique, as the 3D-printed model, being an exact replica of a normal ear, can be duplicated for the trial step before laboratory processing.	According to the authors, the IMM technique is preferred over the DMM technique, as the 3D-printed model, being an exact replica of a normal ear, can be duplicated for a trial step before laboratory processing. Hence, a more predictable outcome can be achieved.	Anterior marginal fit was compromised following the DMM-derived prosthesis and the IMM-derived prototype model. However, following the IMM technique, the duplicated wax pattern was well adapted during the trial session, which improved the final outcome.
Sanghavi, et al., 2018 [49]	The intact ear was scanned and mirrored on the defect side to obtain the 3D-printed model. The model was duplicated into a wax pattern for the trial phase and processed conventionally.	This technique provides an accurate shape and form of the ear, which can be duplicated and processed in a conventional way, thereby making it an economical solution when compared to other 3D printing techniques.	According to the authors, the duplication step can induce errors such as distortion of the wax pattern, which would limit the accuracy and esthetics of the final prosthesis.
Ferreira R, Vives P. 2019 [50]	Custom plates with three locator attachment positions were designed digitally and printed with grade two titanium material. During the surgical procedure, the plates were screwed on the mastoid bone and used to retain ear prostheses without any reported complications.	The use of customized titanium plates can eliminate the problem of limited bone availability for craniofacial implants. This surgical procedure is safe and faster when compared to the placement of implants to retain facial prostheses.	NM
Vijverberg MA et al., 2019 [51]	A total of 31 implants were placed in 12 patients after digital planning and the design of a surgical template. None of these implants failed. GBI displayed positive scores from patients’ responses.	By digitally planning and designing surgical templates, higher accuracy and precise placement of craniofacial implants can be achieved. It further helps anaplastologists design the auricular prosthesis without compromising the retentive attachment position.	The study was from retrospective data; therefore, there might be missing data from patients’ records. Furthermore, Holger’s scores were not mentioned in two cases.
Cevik and Kocacikli. 2020 [52]	The intact ear was mirrored on the defect side. A negative mold was designed digitally and printed for silicone packing. This procedure reduced the fabrication steps, and the fabricated prosthesis fit well on the patient.	This technique saves a number of laboratory and clinical steps by mirroring the intact ear to the defect side and fabricating the mold without conventional wax and flasking procedures.	NM
McHutchion and Aalto. 2021 [53]	A total of five patients were scanned for ear defects, and scan bodies were designed and printed to fit on the implants. The intact ear model was scanned and mirrored on the defect side. Molds were designed and printed for silicone packing to obtain auricular prostheses. Two prostheses fit well, while the remaining prostheses had open margins against adjacent tissues.	This digital workflow can be used as the starting point for future planning and design of facial prostheses, following some improvements.	Two prostheses had a poor fit against the tissues. The third prosthesis fit well in some areas; however, a large gap was noticed along the upper edge of the prosthesis. Test prostheses exerted less pressure on underlying tissues, and surface details were lacking.
Domingue D. et al., 2021 [54]	Craniofacial implants were planned with digital planning software to be placed in the right mastoid location for an auricular prosthesis. Implants were precisely placed at the planned location with no clinically reported complications.	This technique prevents damage to the critical underlying tissues and optimizes the prosthetically driven approach to implant placement with precise angulation and depth.	NM
Unkovskiy et al., 2021 [55]	A silicone auricular prosthesis was printed with various shore A hardness of the silicone. External staining, sealing, and finishing were performed conventionally. The anterior margin was further adjusted with conventional silicone to blend with adjacent skin.	Through proper digital workflow and mirror imaging techniques, the precise size, shape, and position of the auricular prosthesis can be achieved.	The printed silicone prosthesis lacked surface details due to limited printing resolution. Furthermore, grinding with abrasive paper to create a staircase effect further made the silicone surface smooth.
Dashti et al., 2022 [56]	The intact ear was mirrored on the defect side to obtain the exact size and shape of the contralateral ear. Stereolithographic casts, substructures, and milled bars were obtained following virtual planning and design. The wax pattern was copied and processed conventionally to fabricate an auricular prosthesis.	This technique provides a stable working cast without the need for convectional impression steps.	The limitation of this technique was the scanner’s inability to record functional movements. It did not affect the outcome in the present study, but it can be a limitation in cases of excessive tissue movement during function.
Hatamleh MM et al., 2022 [57]	Implants were guided through surgical templates produced by using 3D planning software.	Symmetry is relatively easier to achieve using 3D planning software in cases of bilaterally missing ears.	NM
Heydenrych A et al., 2023 [58]	A surgical template to guide the implants and a template to orient the auricular prosthesis were designed and manufactured with rapid prototyping techniques.	The authors recommend this technique as it eases implant placement, saves time, and helps in the orientation of the auricular prosthesis in relation to the implants.	Implants were deviated in relation to the planned implant position by an average of 4.67 mm. The orientation guide helped overcome the discrepancy in implant position.

## Data Availability

Not applicable for this systematic review.
